# Is there a dose-response relationship? Investigating the functional form between COVID-19 incidence rates and life satisfaction in a multilevel framework

**DOI:** 10.1007/s10902-022-00542-1

**Published:** 2022-06-21

**Authors:** Felix Bittmann

**Affiliations:** Leibniz Institute for Educational Trajectories (LIfBi), Wilhelmsplatz 3, 96047 Bamberg, Germany

**Keywords:** Life satisfaction, COVID-19, Functional form, Incidence, Multilevel, First-difference

## Abstract

While there is plenty of research linking the effects of the global COVID-19 pandemic to a drastic reduction of life satisfaction in the population, there is little information on the functional form of this relationship. Until now, one could suspect that this association is linear and a higher number of COVID-19 infections in a region leads to a continuous decline of satisfaction. However, there are reasons to assume that this interrelation is indeed more complex and deserves further attention. To resolve this question, high-quality panel data of the first wave of COVID-19 from Germany are analysed in a fixed-effect multilevel framework. With information from more than 6,000 respondents (after imputation) nested in 339 federal districts, we estimate linear models with higher-order terms up to the fifth degree of median COVID-19 incidence rates and random intercepts for districts to describe the functional form. The results indicate that even regions with very low incidences are affected and a linear decline of satisfaction is only apparent for rather low incidence levels, quickly reaching a plateau, which is then quite constant, even for higher incidence levels. These findings indicate that at least in rich and industrialized countries like Germany, assuming a strictly linear relation between incidences and change of satisfaction is not appropriate.

## Introduction

The COVID-19 pandemic, which first emerged in early 2020, has disturbed the world like no other global challenge after World War II (Frankema & Tworek, 2020). Virtually all areas of life have been affected and even rich and industrialized countries like Germany were massively transformed by the crisis on various levels (Naumann et al., 2020). To curb the spread of the disease, schools and other institutions of education and care were closed. Workplaces were also often shut down and millions of workers sent home for remote working. Even basic rights like the right of freedom of assembly were curtailed, preventing the meeting of friends and family in the fear of an uncontrollable spread of the virus. As a quickly growing body of international literature reveals, COVID-19 has influenced the quality of life in a negative fashion as various measurements of life satisfaction, well-being or happiness have fallen dramatically (Möhring et al., 2020) while levels of stress, anxiety and depression increased (Dymecka et al., 2021). Since these findings are robust and by now well established in multiple countries and cultures, they lead to follow-up questions. The first one is whether there is a dose-response relationship between the severity of COVID-19 in a given country or region and life satisfaction. For various reasons, one could expect that regions that are hit harder by the pandemic (for example, measured by the standardized number of new COVID-19 cases, the incidence, or the number of deaths due to the disease) register a more severe decrease in satisfaction than less affected regions. Furthermore, what exactly is the functional form of this relationship? Is it linear or are more complex interrelations possible? These open questions deserve more attention to better understand how the pandemic influences satisfaction, which is of greatest relevance for various scientific areas, like social science, health research or economics. Using high-quality longitudinal and regional data from Germany, we investigate these questions in-depth with multilevel analyses. The data were collected directly after the first wave of COVID-19 in Germany and cover the time between May and June 2020 after the first comprehensive national “lockdown”.

## Theoretical expectations

The emergence of COVID-19 in early 2020 influenced the satisfaction of the population in mostly two ways: First of all, there is the disease itself, which entails a rather high mortality rate, especially for older and pre-diseased persons (Baud et al., 2020). In this respect, it is hardly surprising that satisfaction levels fall, since there is obviously a chance of becoming infected and suffering the consequences of COVID-19. As the general hazard of the disease to the population, which is mainly due to the combination of lethality and transmissibility, was quickly recognized, numerous legal regulations and restrictions on public life were put into effect in Germany to prevent its spread and protect the population. These unprecedented and comprehensive regulations came with the restriction of basic rights and brought most of public life to a standstill (Wieler et al., 2020). Workplaces were closed and employees sent home or on short-time work; schools, kindergartens and other educational institutions were closed and children handed over to the care of their parents, who in turn were disrupted in the pursuit of their own careers and other activities. Homes for the elderly and hospitals were isolated, often cutting off contact between relatives and patients. Private contacts were also largely prevented, so that private meetings and visits were reduced to a minimum.

There is no doubt that these restrictions and the associated consequences have had a significant negative impact on the general satisfaction of the population, as a large number of studies from various countries have shown, for example for Germany (Bittmann, 2021; Brandt et al., 2021; Huebener et al., 2020; Möhring et al., 2020; Ravens-Sieberer et al., 2021; Zacher & Rudolph, 2020), the UK (Foa et al., 2020; Shen & Bartram, 2020), Spain (Blasco-Belled et al., 2020), Italy (Maugeri et al., 2020), China (Wang et al., 2021; Zhang et al., 2020) or other countries (Gawrych et al., 2021; Özmen et al., 2021). However, the question arises what this means for the dose-response relationship between the severity of COVID-19 and the effects on satisfaction. For example for Germany, the first “lockdown” from March to May 2020 was enacted on a nationwide level as a consequence of the collaboration between national and federal governments (as each of the 16 German federal states has its own government with special rights and duties). These regulations were independent of local incidences and hence identical for the entire nation, which makes this a period-effect (Kosloski, 1986). Therefore, it is unclear whether one would expect a strong dose-response relationship between satisfaction and local incidence as, for example, restaurants and schools were closed independently of having a high or low local COVID-19 incidence. This is an argument for nationwide similar effects and only weak influences of incidences on satisfaction. However, there are also counterarguments. For example, especially in the first wave in early 2020 as COVID-19 was completely new to the population and the measures unprecedented, in contrast to the following waves, the population was quite sensitive to updates to “the numbers” and local newspapers reported daily new infections and deaths on a regular basis. Also, the higher the incidence, which is a transformed indicator of the share of the local population that is infected, the higher the probability of contracting the disease through other people. This also means that the probability increases that a family member, close friend or neighbour is infected, which probably affects one’s satisfaction. Consequently, there are still arguments which support a stronger dose-response relationship. Hence, at this point, we argue: the higher the incidence in a region, the larger the decrease of satisfaction in that region (Hypothesis 1).

The next logical question is how exactly the function form of this relationship looks like. Is it a linear trend, so that each increase of the incidence leads to lower satisfaction scores? Or is it more complex, as, for example, saturation effects might occur from a certain point on? Given the arguments made before, one could also expect composite effects, which are created through a baseline effect (due to the nationwide “lockdown” regulations) and the dose-response effect, which is due to the locally different number of infected people. Given that these associations might be complex, it is not feasible to give a prediction about the concrete functional form of this relationship, which is also due to the lack of empirical research. Hence, at this point, no hypothesis is formulated. A review of the literature also shows that this question has not been answered in detail yet. One study from the US investigates the consequences of COVID-19 mortality rates on measures of depression and anxiety in April and May 2020 but only looks into linear trends (Le & Nguyen, 2021). The same limitations hold for another, multi-country analysis, which also reports significant linear effects of incidences on various outcomes like happiness (Nguyen, 2021). Some further studies compare high-incidence regions to low-incidence regions, yet have only a few regions included and are therefore quite coarse or only investigate linear trends (Liu et al., 2020; Qiu et al., 2020). One study from Germany compares incidence rates of the 16 federal states, yet only looks into linear trends for the outcome of physical activity (Beck et al., 2021). To our knowledge, there is no comprehensive study available that analyzes the functional form between COVID-19 incidences and some measurement of satisfaction (also happiness, psychological distress or well-being) in a detailed fashion. We attempt to close this knowledge gap by using high-quality German panel data with a fine-grained level of measurement as several hundred different districts are available for multilevel analysis.

Some words on the federal administrative system in Germany, which is a consequence of its highly decentralized character. In total, there are 16 federal states (*Bundesländer*). Each federal state is divided into finer-grained districts (with some different levels for the city-states Berlin, Bremen and Hamburg). On a lower level are cities (*Kreisfreie Städte*, “district-free cities”) and districts (*Landkreise*), which are equivalent to each other from an administrative perspective. The level below are municipalities (*Gemeinden*). Districts correspond to level-3 administrative units in the Nomenclature of Territorial Units for Statistics (NUTS 3). For example, the federal state of Bavaria comprises a total of 2056 municipalities in 2020, which are organized in 71 districts and 25 cities. Larger cities usually have a district of their own and a corresponding district for the adjacent areas.

## Data, sample and methods

### Data and sample

The following analyses make use of the German National Educational Panel Study (NEPS), which is a multicohort-sequence panel study with a focus on the role of education for the life course (Blossfeld & Roßbach, 2019; NEPS Network, 2021).^1^ We utilize starting cohort six, which sampled adults in the first wave of the panel in 2009/10. Since then, participants are surveyed approximately once a year, usually via telephone (CATI) or personally (CAPI). The data used in this study is taken from two survey waves: wave 11, the last regular survey wave that was not affected at all by COVID-19 since the data were collected between September 2018 and April 2019, and the COVID-19 extra survey, an unplanned and spontaneously enacted survey to collect information about the situation of participants during and after the first wave of COVID-19 in Germany, collected between May 13 and June 22, 2020. By doing so we can compare the pre-crisis satisfaction to the post-crisis values in a rather causal fashion.

The realization rate in wave 11 is 88.2% (Steinwede & Aust, 2019); in the COVID-19 extra survey this share drops down to 27.3% (Weiß, 2020), which is drastically below the average NEPS realization rates. The reasons for this low value are manifold: firstly, this was an unplanned extra survey so the participants were not informed about it up until shortly before the survey took place. The mode of the survey was changed to an online and self-administered survey mode, so no human interviewer was present or at the telephone. Probably most importantly, the survey took place in the presence of COVID-19, which induced an overall stressful environment and put a lot of additional burden on the participants, for example, as schools and daycare facilities were closed and children had to be taken care of at home. These are drawbacks for the quality of the analyses, which we attempt to amend with imputation methods introduced further below.

We impose only a few sample restriction criteria. To avoid imputing information for participants who have dropped out much earlier in the survey, only participants were retained who took part in wave 11 of the survey and have valid information on the life satisfaction item and their place of residence since this information is essential to merge the incidence rates.^2^ In addition, after inspecting the data, we removed four districts with very high median incidences (more than 30), which can be considered outliers, thus removing 29 respondents.^3^ After accounting for a few cases that could not be imputed, this gives a final number of 6.402 respondents which are included in the following analyses.

### Operationalization

The dependent variable, overall life satisfaction, is operationalized using this item: “First of all, I would like to ask you some questions about your current satisfaction with various aspects of your life. Please answer on a scale from 0 to 10. ‘0’ means that you are ‚completely unsatisfied’, ‘10’ means that you are ‚completely satisfied’. You can graduate your answer with the numbers [integers] in between. All in all, how satisfied are you with your life at the moment?”. This variable with eleven levels is available in all waves of the survey, including the COVID-19 extra survey. For the analysis of the given research question and overall life satisfaction, the NEPS recommends using this single variable and not generating a compound score. We will use it to trace how satisfaction has changed from wave 11 to the COVID-19 survey. While this single item gives fewer variation than a compound score, it has been successfully analyzed in various other studies, so we believe its reliability and overall quality is high (Bittmann, 2021; Brandt et al., 2021; Möhring et al., 2020).

The second main construct is the COVID-19 incidence rate. These data were taken from official statistics as published by the Robert-Koch-Institute (RKI), which is a German federal government agency and research institute responsible for collecting and publishing the COVID-19 data collected by the 16 German federal states.^4^ We used the raw data on COVID-19 incidences, averaged over the last seven days, so there is one measurement for each district and calendar week available.^5^ This is the standardized incidence, which is computed as follows: Total number of cases minus total number of cases seven days earlier, which is then divided by the total number of inhabitants of the city / district and finally multiplied by 100,000. For example, an incidence of 50 means that 50 new cases per 100,000 inhabitants were registered in the past seven days in a given city / district. These incidences are available for each city / district, starting with calendar week five in 2020 (with some values missing). To compute the median incidence for the relevant time frame, all weeks were selected until the end of the COVID-19 survey by the NEPS, which was until June 22, 2020; this corresponds to including calendar week 25. Over all available data points, separately for each district, the median of all incidences was computed. The median was chosen for several reasons: Firstly, due to the newly established process of the data collection and the difficult situation for the agencies and ministries, especially at the beginning of the pandemic, not all reported numbers are perfectly reliable and it is not uncommon that there are values missing for some weeks.^6^ Secondly, the median is rather robust to outliers so that a very high value in only a single week does not much affect the result, in contrast to the arithmetic mean, which can be driven by a few large values. We believe that it makes more sense from a theoretical perspective to investigate the “average” situation in a given district, which is better represented by the median as it provides a higher temporal stability. Therefore, the median incidence is quite robust when either very high values or very low (or missing) values are present. Consequently, we investigate the effects of median COVID-19 incidence rate in the following analyses.

At this point we want to grant that besides the number of infections, other measurements are also potential indicators of the severity of COVID-19 in a region, for example the number of deaths due to COVID-19. We decided against this measure for mostly two reasons: first, due to various influences, the overall mortality of COVID-19 in Germany in the first wave of the pandemic was rather low and drastically below the average of other comparable countries, which gives this indicator a lesser impact and also a smaller variation between districts. Second, in contrast to diagnosing the presence of the virus in a person, which is a rather objective medical procedure, it is more difficult to attribute a death to COVID-19 (as some people die *from* the disease, while others die *with* it) as the exact causal mechanism of death are sometimes not absolutely clear. Hence, this measurement also comes with another level of measurement error, which might lower its quality.

### Strategy of analysis

As longitudinal data with repeated surveys of the same individuals are available (panel data), it makes sense to take advantage of this design and compute fixed-effect estimates. By doing so, all time-constant covariates are automatically accounted for, like the influence of gender, year of birth or migration status. Even as we can consider the emergence of COVID-19 as a random and external “shock” with unclear causes, rendering the inclusion of classical control variables unnecessary (Bartram, 2021), this design guarantees for a high degree of internal robustness. Some researchers believe that controls are to selected on the basis that they influence the dependent variable (here, life satisfaction). But a more appropriate criterion involves selection also on the basis that the controls are antecedents of the focal *independent* variable. Here the focal independent variable is: regional infection rates. The factors that influenced infection rates in different regions are almost certainly not themselves determinants of life satisfaction. If we include control variables that do not apply this criterion, we risk introduction of bias via ‘overcontrolling’; we might also disturb the functional form that is to be investigated. Additional information on this issue is presented in the limitations below. To be concrete, a first-difference design is chosen, which is numerically equivalent to a longitudinal fixed-effect (FE) analysis since there are exactly two points in time (the pre-crisis survey in 2018/19 and the following extra survey in May and June 2020) (Brüderl & Ludwig, 2015). Not only is this estimator under certain circumstances more efficient than a FE-estimator (Wooldridge, 2010, pp. 315–321), but it is also better compatible with multilevel-analyses, which are of central interest. Hence, for each participant, the change score of life satisfaction is computed where negative values represent a decrease of life satisfaction and positive values an increase. This variable is the dependent variable, the median incidence the independent variable. To account for the spatial clustering of the data, multilevel regressions are computed with random intercepts for each district to account for the fact that different districts are affected differently by the pandemic and that respondents within one district might be more similar to each other than to respondents in other districts. To model the functional form of the relationship, higher-order terms of this variable are added in a stepwise fashion to account for non-linear trends and derive empirically the optimal regression fit. Finally, this allows us to predict the functional form between the incidence and the change of satisfaction. As usually applied to first-difference analyses, the regression intercept (constant) is removed from the equation, forcing the regression line to go through the origin of the coordinate system (Gujarati & Porter, 2010, p. 327). This also makes sense from a theoretical point of view as this means that an incidence of zero (that is the absence of COVID-19) should result in a stable life satisfaction and no change should occur, on average. In mathematical notation, the complete model M5 including all terms can be expressed as follows:

ΔS_ij_ = S_t1, ij_ – S_t0, ij_ = β_1_I_j_ + β_2_I^2^_j_ + β_3_I^3^_j_ + β_4_I^4^_j_ + β_5_I^5^_j_ + u_0j_ + ε_ij_.

The median incidence I only varies by district and hence receives index j, which runs over all districts in the sample; u_0j_ is the district-specific effect on the outcome and epsilon is the level-1 residual term.

To account for the quite large share of missing information on the dependent variable, the data are multiply imputed using MICE (multiple imputation with chained equations) to reduce bias due to selective non-response (Azur et al., 2011). In total, 63.5% of the dependent variable are imputed. As outlined before, there are various reasons why the COVID-19 extra survey in 2020 generated a smaller response-rate than regular surveys. If one believes that this nonresponse is correlated with life satisfaction (for example, people who were extremely stressed by the crisis as they had to take care of their children as schools and other educational institutions were closed), bias can emerge in a complete-case analysis (listwise deletion). By using a large number of potential predictors of life satisfaction from previous waves, which are rather stable over time (like year of birth, migration status or the number of children in the household), this bias can be amended. As strong predictors of life satisfaction are available due to the long-running nature of the panel, we argue that it is feasible to impute the dependent variable (Sullivan et al., 2015). Therefore, we generate 100 complete datasets using the following variables as predictors: life satisfaction in the previous wave, gender, age in 2020, average age in a district in 2020, logged total and monthly after-tax household income, number of persons in the household, number of children younger than 18 years in a household, whether German is the native language of the respondent, the educational level of the respondent, the self-rated health of the respondent in 2020 (rated on a five-point Likert scale where higher integers represent better health), the marital status of the respondent, the employment status of the respondent, the federal state of residence in 2019 (statistics not shown due to data protection regulations of the NEPS) and the median COVID-19 incidence. To further ensure that the data are representative of the overall German population, census-calibrated weights are applied in the following analyses. Descriptive statistics are reported for these variables in the following section in Table 1. All analyses are computed in Stata 16.1 using the additional package *mimrgns* (Klein, 2014). Complete Do-files are available upon request.

**Table 1:** Descriptive statistics

	Mean	SD	Min	Max
Change in satisfaction	-0.96	2.06	-10	10
Median COVID-19 incidence	8.50	5.04	1.10	28.2
Age in 2020	54.7	11.8	34	76
Average age in a district	45.3	1.94	40.9	51.6
Log. monthly post-tax household income	7.98	0.54	4.91	10.6
Self-rated health	3.56	0.79	1	5
Female respondent	0.52	0.50	0	1
German not native language	0.23	0.42	0	1
Number of children per household	0.48	0.91	0	6
Number of people per household	2.56	1.25	1	16
Educational level				
Low (*Hauptschulabschluss*)	0.30	0.46	0	1
Intermediate (*Mittlere Reife*)	0.39	0.49	0	1
High (*Fach-/Abitur*)	0.16	0.37	0	1
Tertiary	0.31	0.46	0	1
Marital status				
Single	0.18	0.39	0	1
Married (or equivalent)	0.70	0.46	0	1
Divorced	0.077	0.27	0	1
Widowed	0.037	0.19	0	1
Employment status				
Regular employee	0.56	0.50	0	1
Public employee	0.030	0.17	0	1
Self employed	0.079	0.27	0	1
Other	0.029	0.17	0	1
Not employed / retired	0.31	0.46	0	1

*Source: NEPS SC6, own computations. Imputed data (M = 100). Census-calibrated weights applied*

## Results

First, descriptive statistics are presented to characterize the sample in Table 1. Since census-calibrated weights are applied, we assume that the resulting sample is an approximation of the overall German population at the time of the pandemic.

**Fig. 1:**
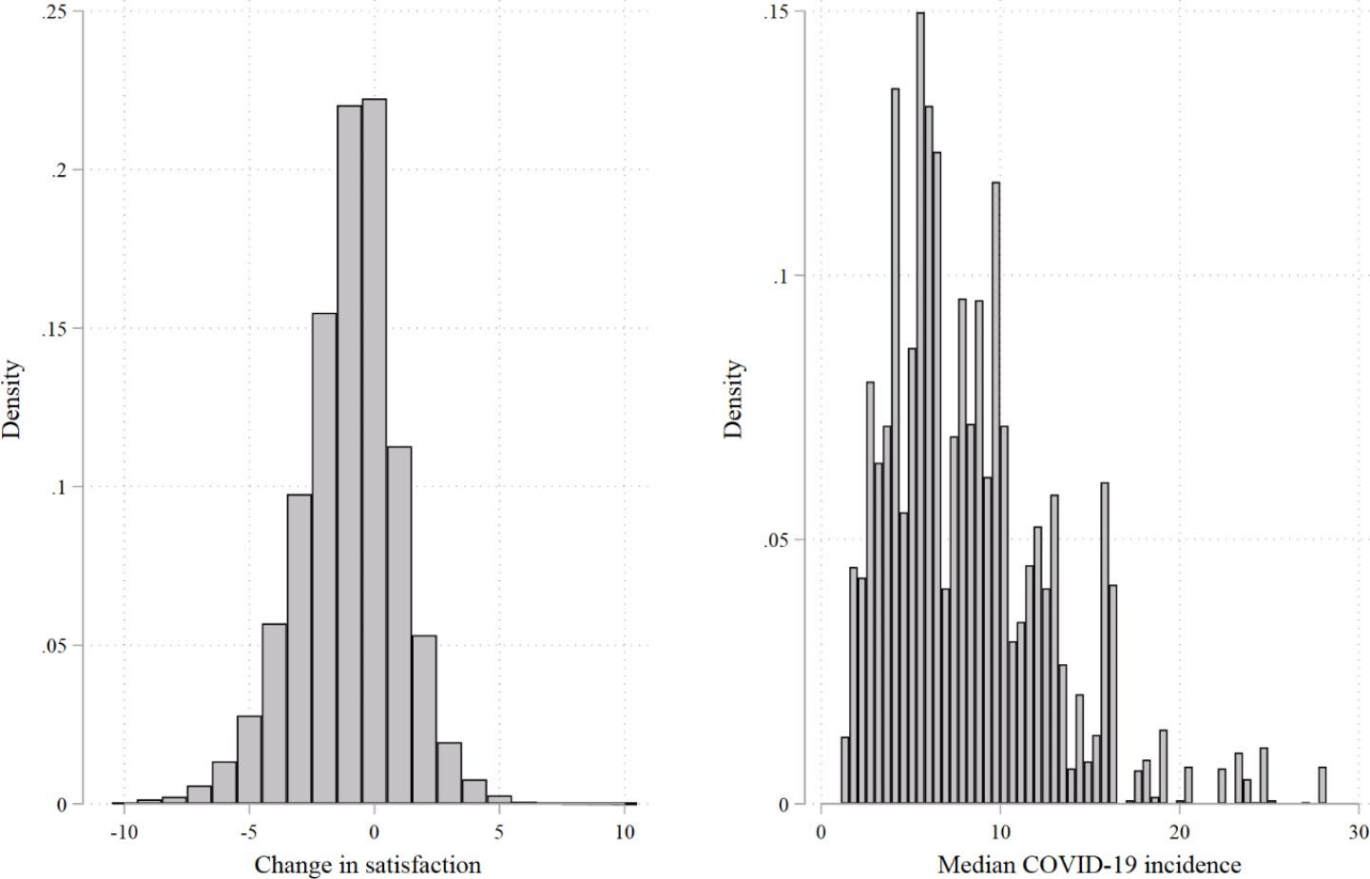
Distribution of the variables change in satisfaction and median COVID-19 incidences

*Source: NEPS SC6, own computations. Imputed data (M = 100).*

**Fig. 2:**
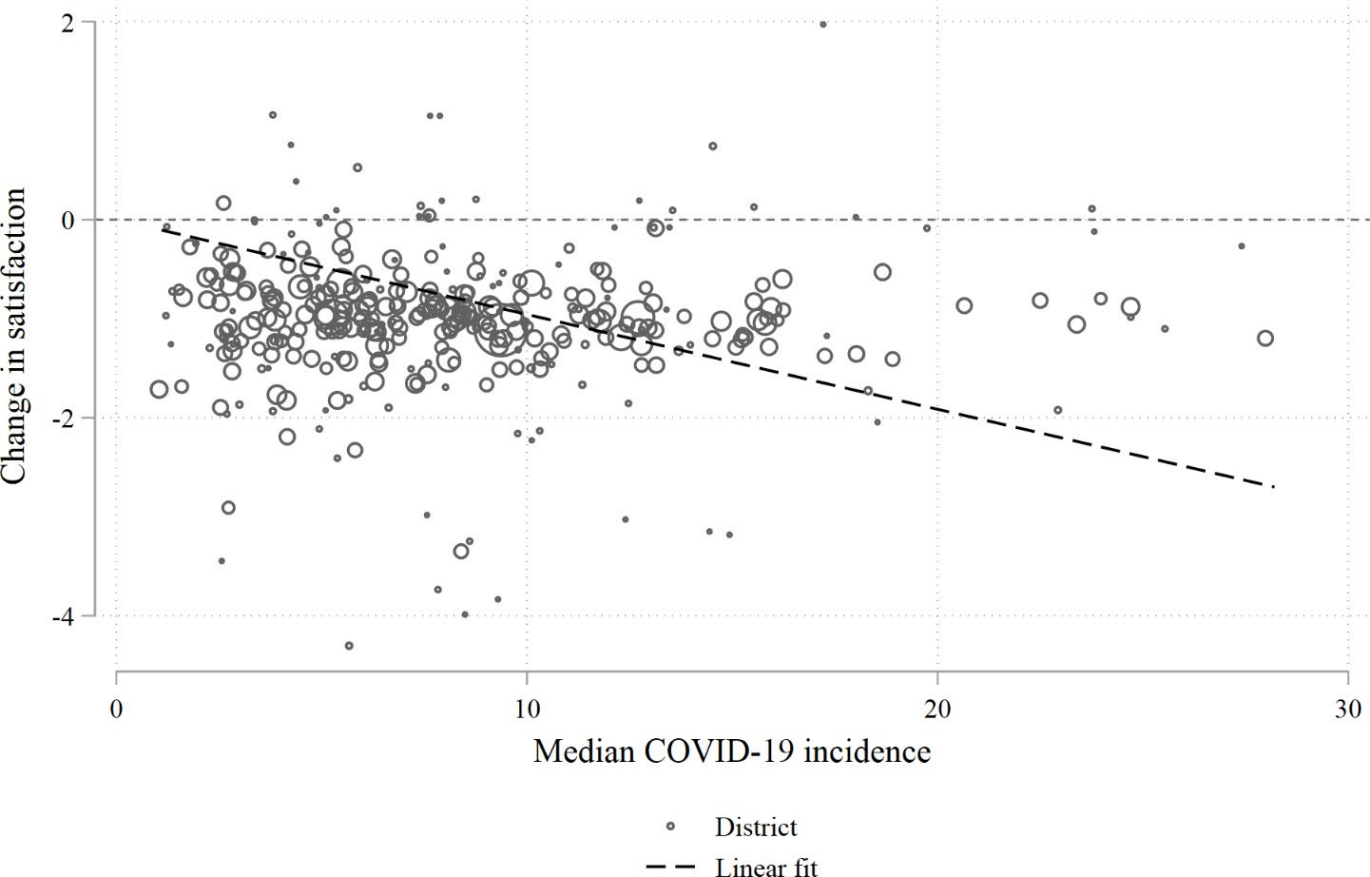
Change in satisfaction on the district level. Source: NEPS SC6, own computations. The size of each circle represents the number of respondents per district. Imputed data (M = 100).

Next, we give a short description of the districts in the sample. In total, there are respondents from 339 districts with an average cluster size of 18.8, a standard deviation of 22.0, a median of 19 and the range being from 1 to 295 (this one large cluster represents the city of Berlin). Figure 1 visualizes the distribution of the two central constructs, the change score of life satisfaction (left side) and the median incidence values (right side). The change score is approximately normally distributed with a mode of zero. This graph already shows that satisfaction has decreased on average after the onset of the pandemic. The distribution of median incidences highlights that most districts have a median incidence between 1 and 10 and only very few incidences are larger than 20. The maximum incidence is 28.2. To give a descriptive overview of the association between change in satisfaction and median incidences, Fig. 2 is created.

Each city or district is represented by a circle with larger circles indicating more respondents. A linear fit regression line is included with the intercept being the origin. Already from this basic figure it becomes clear that a negative association is present and higher incidences correspond to more negative changes of satisfaction. Pearson’s R for this correlation is -0.038 (taking the size of the clusters into account) and statistically significant on the 1% level.

**Table 2:** Multilevel regression results (First-difference estimator)

	M0	M1	M2	M3	M4	M5
Median Incidence		-0.0854^***^	-0.181^***^	-0.344^***^	-0.408^***^	-0.619^**^
		(0.012)	(0.032)	(0.061)	(0.11)	(0.20)
Median Incidence²			0.00660^**^	0.0316^***^	0.0480	0.125^*^
			(0.0020)	(0.0079)	(0.025)	(0.063)
Median Incidence³				-0.000795^***^	-0.00198	-0.0111
				(0.00023)	(0.0017)	(0.0068)
Median Incidence^4^					0.0000251	0.000448
					(0.000035)	(0.00030)
Median Incidence^5^						-0.00000670
						(0.0000045)
Constant	-0.985^***^	-	-	-	-	-
	(0.12)					
Random-effect parameters						
SD (District)	1.19	1.30	1.24	1.19	1.19	1.18
	(0.11)	(0.12)	(0.12)	(0.11)	(0.11)	(0.11)
SD (Residual)	1.70	1.70	1.70	1.70	1.70	1.70
	(0.09)	(0.09)	(0.09)	(0.09)	(0.09)	(0.09)
Observations	6,402	6,402	6,402	6,402	6,402	6,402
Number of districts	339	339	339	339	339	339
AIC	31,161.91	31,204.75	31,181.58	31,165.74	31,166.46	31,164.79
R² (Level 2)	-	-	-0.077	0.003	0.008	0.024
ICC	0.332	0.370	0.348	0.331	0.330	0.326

*Source: NEPS SC6, own computations. Imputed data (M = 100). AIC, R² (Bryk/Raudenbush) and ICC are averaged over all imputed samples. Standard errors in parentheses. Constant not estimated in models which include predictors. Census-calibrated weights applied.* ^*^ *p < 0.05*, ^**^ *p < 0.01*, ^***^ *p < 0.001*

We continue with the main analytical models in Table 2. To do this in a detailed fashion, nested models are computed where higher-order terms of incidence are added step by step. The first model, the baseline, contains no explanatory variables and only gives an overview of the overall change of satisfaction (note that is the only equation that contains the intercept, which represents the average change of satisfaction). The result is about − 0.985, which means that satisfaction has fallen by approximately one point after the onset of the pandemic. We can consider this a huge decrease in overall satisfaction. The next model M1 adds the median incidence as an explanatory variable. As we see, the coefficient is about − 0.0854 and statistically highly significant. This means that when the median incidence in a district increases by one point, satisfaction drops by approximately 0.09 points. This finding is in line with many previous studies and hypothesis 1, which is accepted. In the next models, we keep adding higher-order terms of incidence, that is the squared, cubed, fourth and fifth power of this variable, which is a common procedure to study the functional form between two variables. The more terms are added this way, the better the function approximates the empirical relation. By looking at models M1 to M5, we notice that the coefficients of these terms are mostly statistically significant, which is a sign of good prediction. The overall model fit can be judged using the AIC or R², which is averaged over all imputed samples for each model. The lower the AIC and the higher R², the better the model fit. As these numbers clearly indicate, adding higher-order terms improves model fit as the AIC decreases from M1 to M5. Strictly speaking, M0 with no predictors at all has the “best” fit, which is however not useful as the impact of incidences cannot be investigated with this baseline model. While beyond M3 the addition of even more terms affects the AIC only marginally and even slightly increases the AIC in M4, R² shows that the fit indeed further improves (note that R² is not available in models with only one coefficient due to the way it is computed). While the data are both imputed and use sampling weights, which can affect the interpretation of measures of model fit, there are both theoretical and empirical arguments that M5 is the best model to predict the functional form, hence this model is reported graphically below. While it is difficult to interpret R² in absolute terms, the numbers indicate that incidences only explain little of the overall variance (which is not concerning for the posed research questions). The conclusion is that the functional form between change of satisfaction in a district and median incidence is apparently not linear, otherwise model M5 should not have a better fit than model M1. The ICC is reported for reference yet no clear conclusions can be derived from this measure as the numbers are fairly similar for all models and no patterns emerge

Referring back to the original research question, we can now describe the functional form between incidences and satisfaction in detail? Theoretically, model M5 gives us all the information as we can use the coefficients to predict satisfaction for arbitrary values of the incidence which allows us to describe the functional relationship. We will do this in a convenient fashion and plot the results including 95% confidence bands, using the range from 1 to 25 (since above, only very few cases are available and confidence bands are extremely broad).

**Fig. 3:**
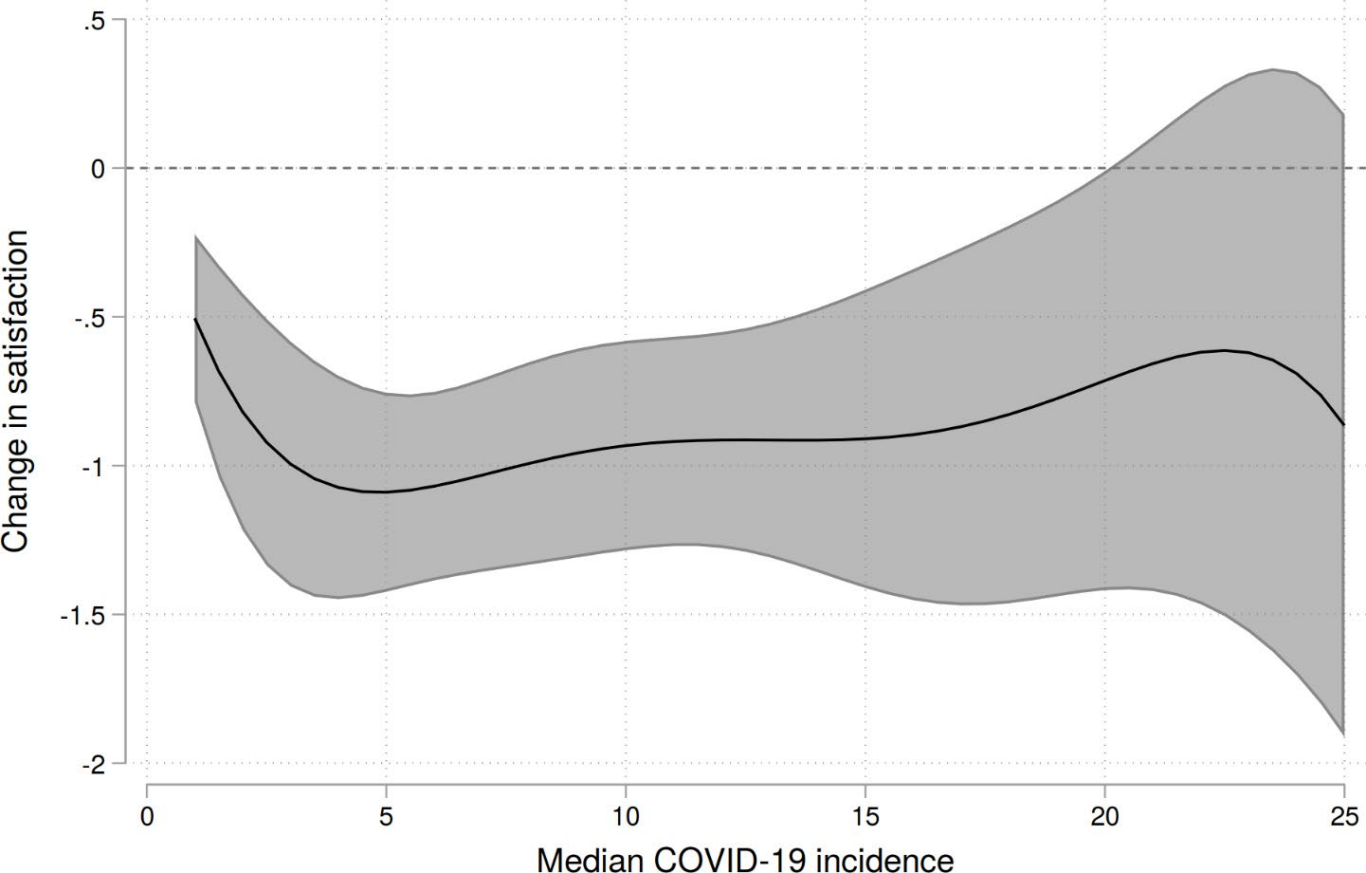
Predicted change in satisfaction by model M5. Source: NEPS SC6, own computations. Imputed data (M = 100). Census-calibrated weights applied.

The predictions are shown in Fig. 3. First, we notice that even in the regions with very low incidences, satisfaction has fallen sharply by about 0.5 points. We observe a quite linear decrease in the range of incidences from 1 to about 5, after that, the curve quickly flattens and even turns back into less negative values. However, as the confidence bands are very broad, due to the low number of observations with rather high incidences, the uncertainty is quite large. Although we observe these fluctuations of the curve, it makes sense to assume a rather constant development due to the quite broad confidence bands. This has also been indicated by Fig. 2, where only very few observations fall below − 2, even in regions with the highest incidence rates. It must also be made transparent that even a fifth-order polynomial is not perfectly able to capture the empirical relationship so we would expect some minor ups and downs from a mathematical point of view. This trend is stable, only the confidence band increases as the number of available data points for high levels of incidence decrease.

Finally, we computed some robustness checks to investigate the stability of the findings. First, instead of using median incidences, the averages (arithmetic means) were tested as well. As the high correlation between these two statistics already indicates (Pearson’s R = 0.76), findings are rather similar and the functional forms are highly comparable. Second, computations were repeated with not imputed data (listwise deletion applied, N = 2,335) to test the influence of the imputation procedure. Again, only little differences between the findings arise, supporting the claim that there is no bias due to the imputation.

## Discussion

As the results indicate, higher incidences of COVID-19 are associated with a significant decrease of life satisfaction. This finding makes sense in the light of theoretical expectations and previous publications. The part that is of greatest interest is the modeling of the functional form between median incidences in a region and satisfaction. The models also indicate that higher-order terms of median incidences increase the model fit and the overall functional form between incidences and satisfaction is not strictly linear. When describing the form in detail, we observe a baseline effect of about 0.5 points, even in the least affected regions. Referring back to our theoretical expectations, this makes sense as most restrictions, like closing of workplaces, schools or other institutions of daily life were imposed on a nationwide level, hence affecting all people. Therefore, even when there are locally almost no infections present, satisfaction is still reduced. Beyond this point a quite linear decline is visible, which means that higher incidences lead to a decrease in satisfaction. However, this form quickly reaches a rather stable plateau of an incidence between 5 and 15. After that point, satisfaction is no longer affected by rising incidences with the exception of some, probably random, minor fluctuations. While the certainty of the results decreases due to fewer data points, we can be rather confident that no major drops in satisfaction are likely.

These results are highly interesting for multiple reasons. Firstly, they indicate that a nationwide effect of the crisis is detectable, even in the near absence of local infections. Apparently, this share of the effect is due to regulations and consequences of the pandemic, which could also be framed as “byproducts” of the disease itself. One could hypothesize that without these very strict and unprecedented measures, satisfaction would have been higher in the least affected regions. Of course, this is not a recommendation or means that the restrictions were inappropriate, yet one could speculate that a finer-grained level of enacting these measures, that is, imposing them only in regions with higher incidences, would have spared some regions decreases in satisfaction. Maybe even more interesting is that after a certain threshold, rising incidences are not affecting the satisfaction any longer but a stable plateau is quickly reached. Apparently, higher not always means worse after a certain level. On the one hand, this is surprising as the higher the number of infections, the higher the probability to become infected or witness the infection of friends and relatives, on average, which should further decrease the satisfaction. On the other hand, there is probably a psychological limit of how much this fact can influence satisfaction. It is well known that "more" not always means "better", for example for income, where after a certain threshold happiness does not increase further (Jebb et al., 2018). Potentially, similar mechanism might be present in the case of COVID-19.

Finally, the limitations of the analyses should be discussed. First, as already mentioned before, the realized NEPS sample is not perfectly representative of the overall population due to dropouts. To combat this issue, multiple imputation was conducted and census-calibrated weights were applied. Still, minor differences to the overall population might exist, which must be kept in mind when generalizing the results. Second, due to a rather large number of unit-nonresponse in the COVID-19 extra survey, a large share of information was missing, which increases the uncertainty of the results. As indicated by additional complete-case analyses, we do not believe that there is much influence by the imputation itself. From a theoretical point of view, if one believes that respondents who were most negatively affected by the consequences of the pandemic were not able to participate in the survey, all estimates are conservative and the real decrease due to COVID-19 of satisfaction would be even larger. Following this logic, all results reported therefore probably amount to upper bounds and might be even more severe if all respondents would have participated. Third, the reliability of the two main constructs should be critically acknowledged. Life satisfaction is derived from a single item and it is generally known that constructs like *satisfaction*, *well-being* or *happiness* are not straightforward to measure as there are many, often diverging definitions. However, we believe that the kind of operationalization is well-established and our findings are similar to various other published results, as indicated by a review of the literature. The second construct, median COVID-19 incidence, is defined mathematically, yet the obstacle is obtaining accurate numbers, even in highly developed countries like Germany. We believe that the deviation to the true values should not be too large and there are no other or more reliable sources than the numbers reported by the RKI. In addition, the process of computing median incidences should also contribute to a higher temporal stability of the COVID-19 situation in a given district. Fourth, the data give an impression of the situation of the first wave of COVID-19 in Germany in early 2020. While this is clearly of great relevance, findings might be different when the following “lockdowns” and waves of the pandemic were to be analyzed as one could suspect that habituation effects might occur as the novelty of the situation changes. In the absence of suitable data, these questions must be considered in the future. Fifth, as always with regional data, spillover effects can occur, especially for people living close to the borders of a district or city as they might also be affected by adjacent districts. Sixth, results might be biased if there are factors present that change between the time of the first survey in 2019 and the onset of the pandemic on the regional level that confound incidences. As a first-difference regression was used, this concerns only *changes* but not stable difference between regions. Since the time-span is relatively short between the two survey waves, major changes on the macro level are unlikely. Yet, as these can never be ruled out completely, this caveat holds if one wants to interpret the results as causal.

## Conclusions

This study contributes to the growing body of literature investigating the consequences of the global COVID-19 pandemic for quality of life and well-being. While the first finding is that satisfaction is affected negatively by the first wave of COVID-19 in Germany, our results also indicate that this functional form is not linear but more complex. Apparently, larger median incidences in a region do not come with a linear decline of satisfaction but a stable plateau is quickly reached. We believe that this finding is of interest as it gives a better understanding of the detail effects of the pandemic and its consequences for the individual well-being. It also shows that studies which only report a single number or coefficient for the effect must be careful, for example when carelessly interpolating in a linear fashion, which might lead to an overestimation of the impact. Therefore, we advise to be thoughtful with such interpretations and look into the relationship in detail. This is a reminder that researchers of happiness should always be aware of non-linear trends and check for them empirically, which can be achieved by using quadratic terms in regression models or simply by using graphical means. Probably, happiness and satisfaction are prone to saturation or ceiling effects given some special psychological mechanisms. Therefore, researchers studying happiness and related constructs should be especially attentive and think about non-linear forms and relationships. Replicationstudies from other countries or different waves of the pandemic are highly welcome to assess the robustness and overall (temporal and spatial) validity of our findings.

## Footnotes

1 This paper uses data from the National Educational Panel Study (NEPS): Starting Cohort Adults, doi:10.5157/NEPS:SC6:12.0.0. From 2008 to 2013, NEPS data was collected as part of the Framework Program for the Promotion of Empirical Educational Research funded by the German Federal Ministry of Education and Research (BMBF). As of 2014, NEPS has been carried out by the Leibniz Institute for Educational Trajectories (LIfBi) at the University of Bamberg in cooperation with a nationwide network.

2 We also updated some information with data from wave 12 (which were collected between September 2019 and March 2020) for information on rather objective variables, like the number of persons living in the household or the place of residence. We do not believe that this procedure introduces bias since more than 80% of all respondents of this wave were surveyed in 2019 in the complete absence of COVID-19 and the critical item of satisfaction is not updated but taken from wave 11 only for the baseline measurement.

3 Our initial analyses show that these outliers influence the estimation of the functional form to a large extent in the tails of the prediction, which does not make sense from a theoretical point of view and gives very large confidence bands. We believe that these districts should therefore regarded outliers which do not capture the “typical” relation between COVID-19 and satisfaction. Since the overall number of affected participants is also very small, the stability of these findings is probably also low.

4 This process is as follows: whenever a patient is diagnosed with COVID-19, the responsible therapist (general practitioner or doctor in a hospital) is required to report this case to the local public health department, which collects all data from a city or district. This information is then summarized and relayed to the federal health ministry. Finally, each of the 16 federal ministries sends the aggregated data to the RKI which publishes the all-German numbers regularly.

5
https://npgeo-corona-npgeo-de.hub.arcgis.com/.

6 Still, we would like to acknowledge that the numbers reported in Germany are usually of a very high quality in a multinational comparison (Farhadi, 2021).
